# Supplementary material for: Decellularized Matrix Induced Spontaneous Odontogenic and Osteogenic Differentiation in Periodontal Cells
Source: Biomolecules. 2023 Jan 6;13(1):122. doi: 10.3390/biom13010122 (PMC9855832; doi:10.3390/biom13010122)
Supplement: Supplementary file 1 [file biomolecules-13-00122-s001.zip › biomolecules-2048369-supplementary.pdf]

Supplementary Material

Table S1. Data of morphometric analysis of expression of osteocalcin in periodontal cells cultured on decellularized scaffolds under 2D conditions for 14 days**.**

Anova: Two-Factor with Replication

SUMMARY	PDLSCs	PSCs	PDLSCs+PSCs	Total
dTM				
Count	6	6	6	18
Sum	557.0224	558.6011	559.1664	1674.79
Average	92.83707	93.10019	93.19441	93.04389
Variance	14.30319	2.112903	4.38457	6.142054
dPDL				
Count	6	6	6	18
Sum	498.7494	499.7705	499.2014	1497.721
Average	83.1249	83.29508	83.20023	83.20673
Variance	24.25766	5.104121	46.21965	22.23497
dTM+dPDL				
Count	6	6	6	18
Sum	528.8519	523.9058	525.213	1577.971
Average	88.14198	87.31764	87.53551	87.66504
Variance	5.443573	5.01808	32.10998	12.64987
Total				
Count	18	18	18	
Sum	1584.624	1582.277	1583.581	
Average	88.03465	87.9043	87.97671	
Variance	29.5944	20.74668	42.05729	

ANOVA

Source of Variance	*SS*	*df*	*MS*	*F*	*P-value*	*F crit*
Sample (Type of Matrix)	873.4685	2	436.7343	28.28717	**0.00000001**	3.204317292
Columns (Type of Cells)	0.153551	2	0.076776	0.004973	0.995040156	3.204317292
Interaction	2.534966	4	0.633741	0.041047	0.996683086	2.578739184
Within	694.7687	45	15.4393			
Total	1570.926	53				

Table S2. Data of morphometric analysis of expression of osteocalcin in periodontal cells cultured on decellularized scaffolds under 3D conditions for 14 days.

Anova: Two-Factor with Replication

SUMMARY	PDLSCs	PSCs	PDLSCs+PSCs	Total
dTM				
Count	6	6	6	18
Sum	591.1488	592.9355	593.4843	1777.569
Average	98.5248	98.82259	98.91404	98.75381
Variance	0.165595	0.208273	0.443267	0.269575
dPDL				
Count	6	6	6	18
Sum	579.8216	580.399	580.6771	1740.898
Average	96.63694	96.73316	96.77952	96.71654
Variance	1.988622	1.956386	1.325067	1.553756
dTM+dPDL				
Count	6	6	6	18
Sum	584.0999	586.5875	586.9262	1757.614
Average	97.34999	97.76458	97.82103	97.6452
Variance	0.58151	0.508022	3.145786	1.292382
Total				
Count	18	18	18	
Sum	1755.07	1759.922	1761.087	
Average	97.50391	97.77345	97.83819	
Variance	1.446115	1.556542	2.249521	

ANOVA

Source of Variance	*SS*	*df*	*MS*	*F*	*P-value*	*F crit*
Sample (Type of Matrix)	37.45146	2	18.72573	16.32658	**0.0000047**	3.204317292
Columns (Type of Cells)	1.131536	2	0.565768	0.493282	0.61388227	3.204317292
Interaction	0.222939	4	0.055735	0.048594	0.995401387	2.578739184
Within	51.61263	45	1.146947			
Total	90.41857	53				

Table S3. Data of morphometric analysis of expression of DSPP in periodontal cells cultured on decellularized scaffolds under 3D conditions for 14 days.

Anova: Single Factor

SUMMARY

Groups	*Count*	*Sum*	*Average*	*Variance*
PDLSCs	7	174.9657	24.99509	15.56635
PSCs	7	160.9187	22.98839	6.429955
PDLSCs+PSCs	7	189.8346	27.11923	27.85641

ANOVA

Source of Variance	*SS*	*df*	*MS*	*F*	*P-value*	*F crit*
Between Groups	59.73957	2	29.86978	1.797482	**0.194213874**	3.554557146
Within Groups	299.1163	18	16.61757			
Total	358.8559	20				

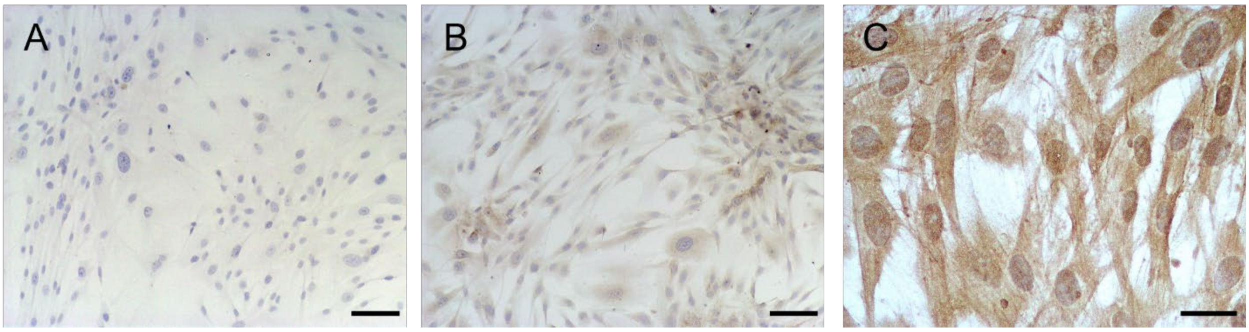

Supplemental Figure S1. Immunocytochemical staining of periodontal cells. The positive cells have brown color. The nuclei were counterstained with hematoxylin: (a) No staining of PSCs for OPN; Scale bar, 50 μm; (b) Weak PSCs staining for CD73; Scale bar, 50 μm; (c) Strong staining of PDLSCs for STRO-1; Scale bar, 200 μm.

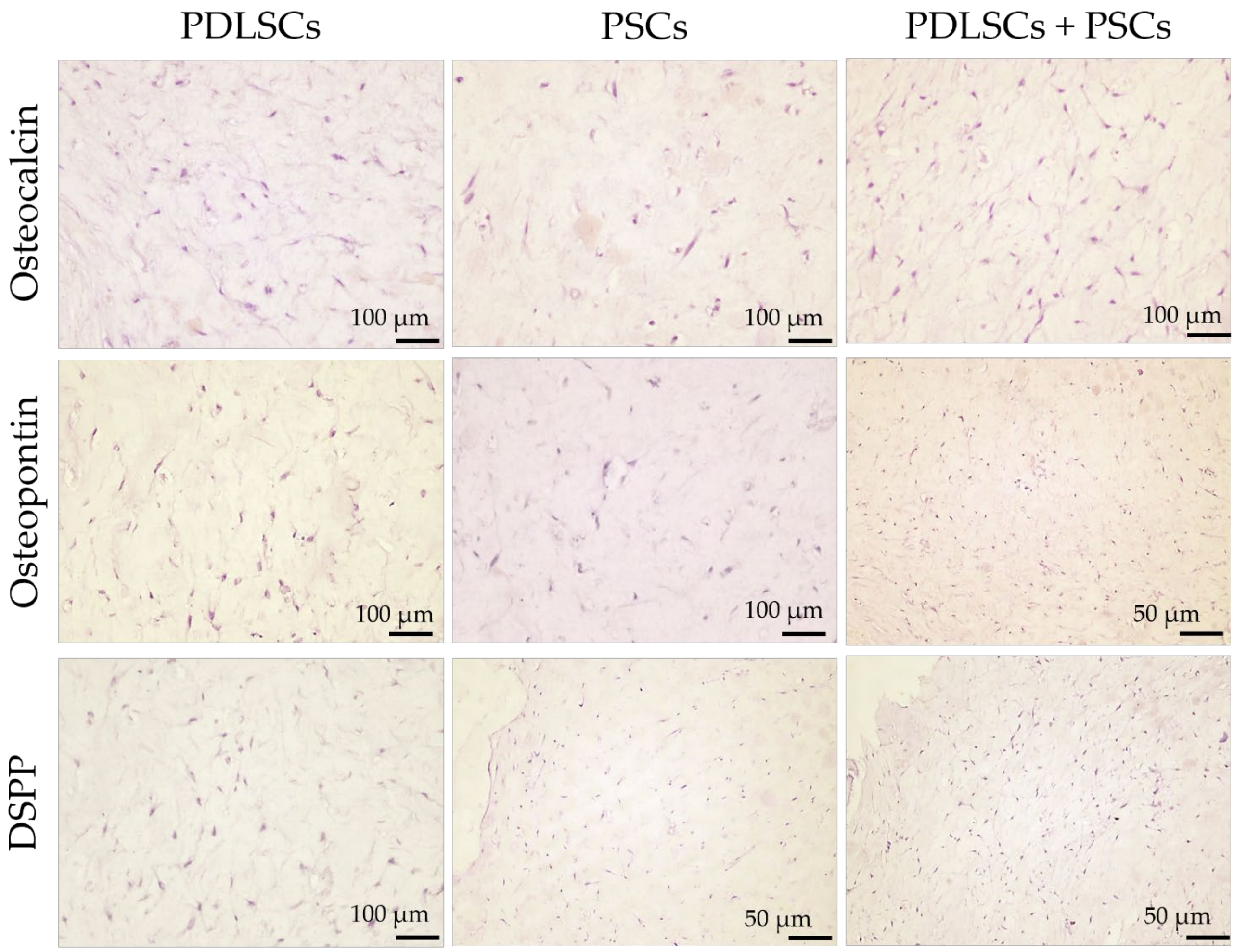

Supplemental Figure S2. Negative control. Immunocytochemical staining for osteogenic and odontogenic differentiation markers in periodontal cells cultured without decellularized scaffolds under 3D conditions for 14 days. The nuclei were counterstained with hematoxylin.
